# Hyaluronan Hydrogels: Rheology and Stability in Relation to the Type/Level of Biopolymer Chemical Modification

**DOI:** 10.3390/polym14122402

**Published:** 2022-06-14

**Authors:** Annalisa La Gatta, Emiliano Bedini, Maria Aschettino, Rosario Finamore, Chiara Schiraldi

**Affiliations:** 1 Department of Experimental Medicine, School of Medicine, University of Campania “Luigi Vanvitelli”, Via L. De Crecchio 7, 80138 Naples, Italy; maryaschettino75@gmail.com (M.A.); rosario.finamore@unicampania.it (R.F.); chiara.schiraldi@unicampania.it (C.S.); 2 Department of Chemical Sciences, University of Naples Federico II, Via Cintia 4, 80126 Naples, Italy; ebedini@unina.it

**Keywords:** hyaluronan, crosslinking, rheology, enzymatic degradation

## Abstract

BDDE (1,4-butanediol-diglycidylether)-crosslinked hyaluronan (HA) hydrogels are widely used for dermo-aesthetic purposes. The rheology and stability of the gels under physiological conditions greatly affect their clinical indications and outcomes. To date, no studies investigating how these features are related to the chemistry of the polymeric network have been reported. Here, four available HA-BDDE hydrogels were studied to determine how and to what extent their rheology and stability with respect to enzymatic hydrolysis relate to the type and degree of HA structural modification. ^1^H-/^13^C-NMR analyses were associated for the quantification of the “true” HA chemical derivatization level, discriminating between HA that was effectively crosslinked by BDDE, and branched HA with BDDE that was anchored on one side. The rheology was measured conventionally and during hydration in a physiological medium. Sensitivity to bovine testicular hyaluronidase was quantified. The correlation between NMR data and gel rheology/stability was evaluated. The study indicated that (1) the gels greatly differed in the amounts of branched, crosslinked, and overall modified HA, with most of the HA being branched; (2) unexpectedly, the conventionally measured rheological properties did not correlate with the chemical data; (3) the gels’ ranking in terms of rheology was greatly affected by hydration; (4) the rheology of the hydrated gels was quantitatively correlated with the amount of crosslinked HA, whereas the correlations with the total HA modification level and with the degree of branched HA were less significant; (5) increasing HA derivatization/crosslinking over 9/3 mol% did not enhance the stability with respect to hyaluronidases. These results broaden our knowledge of these gels and provide valuable information for improving their design and characterization.

## 1. Introduction

**Figure 1:**
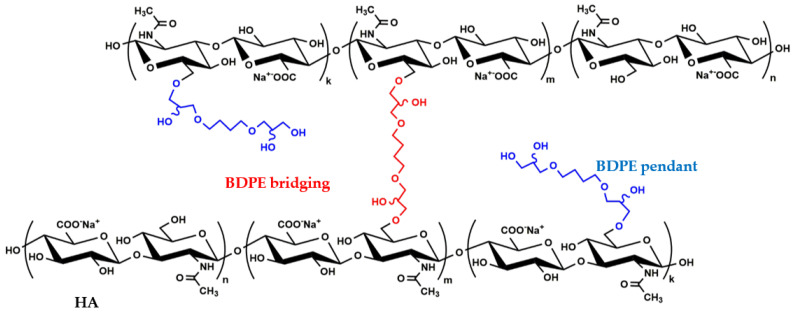
The chemical structure of the HA network formed via the reaction with BDDE. The two resulting 1,4-butanediol di-(propan-2,3-diolyl) ether (BDPE) derivatives are indicated, with BDPE bridging/crosslinking two HA disaccharide units (BDPE bridging, BDPE crosslinking, or double-linked BDPE) in red and BDPE with one side anchored to HA (BDPE pendant; BDPE branching; mono-linked BDPE) in blue. For the sake of simplicity, only the primary hydroxyl functions involved in BDPE linking are depicted, as they are the most reactive and, therefore, the most likely to be modified; nonetheless, the presence of derivatization at any of the secondary hydroxyls cannot be ruled out. The disaccharide units crosslinked by BDPE will be referred to as crosslinked HA_d_; the disaccharide units bearing BDPE pendant will be referred to as branched HA_d_.

Due to their unique combinations of biological and biophysical properties, hyaluronan (HA)-based hydrogels are broadly applied for dermal tissue wound healing, engineering, and aesthetic treatments [1]. Specifically, the hydrogels obtained through the reaction of HA with 1,4-butanediol-diglycidyl ether (BDDE) are widely used for dermo-aesthetic purposes. Intradermal injections for skin rejuvenation represent the most broadly used treatment option. Commercially available gels are variously concentrated suspensions of HA–BDDE networks in a physiological solution. They can differ according to the degree of HA modification by BDDE. Further, they are known to contain two different 1,4-butanediol di-(propan-2,3-diolyl) ether (BDPE) functionalizations (Figure 1). 

In fact, part of BDDE reacts with HA at both ends, effectively bridging two disaccharide units (BDPE bridging) and forming crosslinked HA (Figure 1). Another portion of the di-epoxide reacts with water/hydroxide at one end and with the polymer at the other, thus installing only an ether-linked appendage to HA (BDPE pendant) and forming branched HA (Figure 1) [2,3,4,5].

The modification of HA by BDDE endows it with key features for various applications. Unlike the linear polymer, the crosslinked network is able to swell in an aqueous solution without dissolving, rheologically behaves as a gel-like material (with the storage modulus exceeding the loss modulus and with a slight dependence of the moduli on frequency), shows a viscosity that decreases with the shear rate under flow conditions, and is less sensitive to degradation by hyaluronidases [1,6]. These features are at the basis of a gel’s overall performance, as they are responsible for its hydration, projection (filling effect), injectability, and longer permanence in tissues compared to linear HA. The rheology of the final formula is key for its clinical designation. As the storage modulus is a measure of the gel’s resistance to external forces and its ability to retain its shape, stiffer gels (high-G′ gels) are suited for restoring lost tissue volume (lifting support) and are designated for injection into deeper layers of skin; more deformable preparations (lower-G′ gels) are intended for injection into more superficial dermal layers in order to enhance face contours and for a skin-boosting effect. Studies providing rheological and other biophysical data (swelling, sensitivity to enzymatic hydrolysis, cohesivity, and so on) for clinically available HA–BDDE gels have been intensifying. By allowing the comparison of available gels in terms of certain parameters, they assist clinicians in the selection of the most appropriate products for the specific clinical needs. Fewer studies aiming at properly ascertaining the complexity of the rheological behaviors of these products have been carried out, with Ilyin et al. describing, for the first time, to the best of our knowledge, the mechanical behavior of these gels after dilution in a physiological medium. This represents an aspect that should be further explored in the science of HA-based dermal treatments to indicate gels’ behaviors when interacting with surrounding tissues [6].

Gel rheology certainly depends on the degree of HA derivatization—with different effects expected for the two types of modification—and on the biopolymer concentration (i.e., the higher the HA modification and concentration, the stiffer the gel is expected to be). 

However, to the best of our knowledge, a quantitative analysis establishing this correlation, which would be valuable for tailoring gel design towards specific performance, has not been carried out so far. In fact, the biophysical parameters provided for clinically available gels have not been related to level/type of chemical modification of HA [3,7,8,9,10,11,12,13,14,15,16,17,18,19,20,21,22,23,24,25,26,27,28]. 

Conversely, characterization studies of the structural modifications obtained after the reaction of HA with BDDE have been performed to a lower extent [2,3,4,5,9,12,19,20,29,30,31]. Only some of them have discriminated between the BDPE-bridging and BDPE-pendant moieties. Furthermore, these analyses were mostly performed for homemade gels, and the acquired chemical data have never been considered in relation to hydrogel rheology [3,4,5,12,19,31]. 

To fill this gap, four commercially available HA–BDDE hydrogels with the same clinical indications as volumetric dermal fillers were investigated here for the determination of their amounts of crosslinked and branched HA, stiffness (G′), and overall elasticity (tan δ). Their sensitivity to enzymatic degradation was also studied as another key parameter in view of their application, and it was influenced by the HA modification level. The collected data were then examined to verify whether and how gel rheology and stability are correlated with the type and degree of HA modification. The rheology was also unconventionally studied during the hydration of the gel (progressive gel dilution) in a physiological medium so that it would, thus, better resemble in vivo conditions. Information that will be useful for the improvement of the design and characterization of this type of gel is expected.

## 2. Materials and Methods

### 2.1. Materials

Restylane Lift (R_L_) is distributed by Galderma S.P.A. Juvederm Voluma (J_V_) is distributed by Allergan S.P.A. (Pringy, France). Teosyal RHA4 (T_RH4_) is distributed by Teoxane SA (Geneva, Switzerland). Aliaxin SV (A_SV_) is distributed by IBSA Farmaceutici Italia srl (Milan, Italy).

These are BDDE-crosslinked HA hydrogels that are intended for use as dermal fillers. According to the patented protocols of production, all of the gels were produced from high-molecular-weight HA (≥1000 kDa) with J_V_ using an additional 300–750 of kDa HA. However, this reaction requires high pH values in addition to high temperatures (50–60 °C), and, under these conditions, as demonstrated elsewhere [30], a huge amount of HA depolymerization accompanies the reaction, making the HA chains similar in length while undergoing crosslinking.

The HA concentration values reported in the leaflets for the various gels were the following: 20 mg/mL (R_L_, J_V_), 23 mg/mL (T_RH4_), and 25 mg/mL (A_SV_) [32,33,34,35]. R_L_, J_V_, and T_RH4_ also contained 0.3% lidocaine. These concentration values take into account both the water-insoluble HA–BDDE network, which is the main component of the formula, and a water-soluble HA fraction (4.6–10.4 mg/mL). Previously performed SEC–TDA (Size Exclusion Chromatography–Triple-Detector Array equipment by Viscotek, Malvern Panalytical Ltd, Malvern, UK) analyses revealed that the soluble fractions consisted of chains with M_w_ in the range of 100–230 kDa and M_w_/M_n_ in the range of 2.4–2.9, with T_RH4_ presenting an additional 1100 kDa non-modified HA fraction. It is key that the gels were comparable in terms of their water-insoluble HA–BDDE concentration, which is the main factor responsible for their typical rheological behavior [26]. 

Bovine testicular hyaluronidase (BTH (EC 3.2.1.35)) in a salt-free lyophilized powder was purchased from Sigma-Aldrich S.R.L. (Milan, Italy) (cat. N. H3884). 

Dulbecco’s phosphate buffered saline (PBS) without calcium and magnesium was purchased from Lonza Sales Ltd., Switzerland (cat. N. BE17–516F). 

### 2.2. NMR Analyses

**Figure 2:**
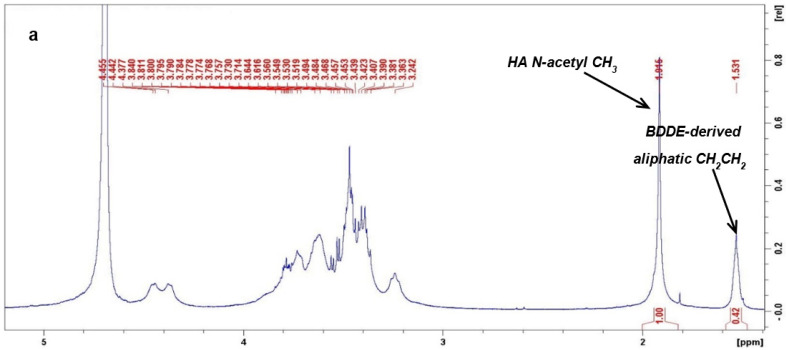

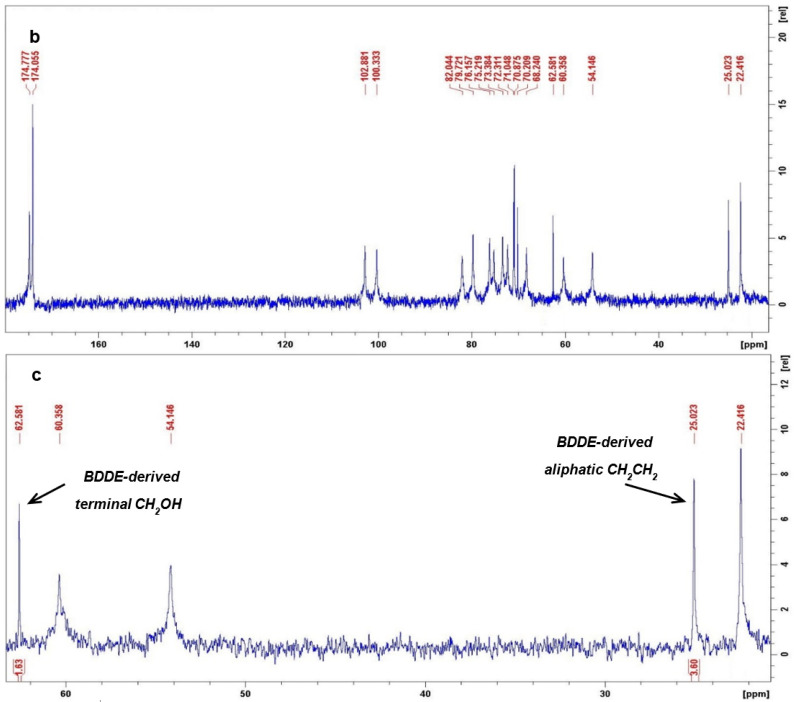
Representative NMR spectra. (**a**) ^1^H-NMR (400 MHz, 300 K, D_2_O) and (**b**) full and (**c**) zoomed ^13^C-NMR (400 MHz, 300 K, D_2_O) spectra of the ASV sample. The signals used for the quantitative determinations are indicated.

The samples analyzed with NMR were the following: Aliaxin SV lot no. 017I2-SV/A, Juvederm Voluma lot no. VB20A80412, Restylane Lift lot no. 15585–1 + lot no. 16222–1, and Teosyal RHA 4 lot No. TPUL-183424B6000. The samples were chemically hydrolyzed prior to the NMR analyses. Hydrolysis was carried out under acidic conditions, as previously reported for these types of hydrogels, but with modifications [29,36]. Briefly, 2–4 mL of each gel was diluted to a final HA concentration of 4 mg/mL in 0.01 M HCl. Suspensions were kept under stirring (800 rpm) at 70 °C for 72 h to observe complete gel dissolution. The samples were then neutralized by adding 0.038 M Na_2_HPO_4_ and then lyophilized. The dried samples were dissolved in D_2_O (600 μL), and the analyses were performed using a Bruker Avance III HD (^1^H: 400 MHz, ^13^C: 100 MHz) instrument (Billerica, MA, USA) at 300 K. Quantitative ^1^H-NMR spectra were measured using a recycle delay (d1) value of 30 s, a number of scans (ns) equal to 16, and a line-broadening factor of 0.3 Hz. Quantitative ^13^C-NMR spectral data were processed using the data analysis packages integrated into the Bruker TopSpin^®^ 4.0.5 software. Representative ^1^H-NMR and ^13^C-NMR spectra for the samples are reported in Figure 2. Spectra for all of the samples tested are reported in the Supplementary Material (Figure S1).

The total BDPE per disaccharide unit (BDPE/HA_d_, molar %) (A) was quantified from the ^1^H-NMR spectra through the relative integration of the signal at 1.56 ppm relative to the aliphatic (CH_2_)_2_ moiety of the BDDE-derived linker with respect to the signal at 1.95 ppm relative to the N-acetyl moiety of the glucosamine units of HA [5,29,36]. 

The amount of BDPE pendant per disaccharide unit (BDPE pendant/HA_d_, molar %) (B) was quantified from the ^13^C-NMR spectra through the relative integration of the signal at 62.6 ppm relative to the -CH_2_OH moiety of the BDDE-derived linker with one side anchored to HA (BDPE pendant) with respect to the signal at 25.1 ppm relative to the N-acetyl moiety of the glucosamine units of HA [5]. 

From (A) and (B), the following parameters were derived:

(C) BDPE bridging/HA_d_ (mol% ) = (A) − (B);
(D) BDPE bridging/total BDPE (mol%) = (C)/(A) × 100;
(E) BDPE/HA_d_ (wt%) = (A) × [(MW BDPE) /(MW HA_d_)],

where MW BDPE = 204.3 g/mol and MW HA_d_ = 401.2 g/mol;

(F) Total BDPE in the gel (wt%) = [HA wt% in the gel × E] /100,

where the HA wt% in the gel = HA (mg/mL)/ 1000 mg gel × 100 (gel density = 1 g/mL);

(G) Crosslinked HA_d_/HA_d_ (mol%) = (C) × 2;
(H) Branched HA_d_/HA_d_ (mol%) = (B);
(I) Modified HA_d_/HA_d_ (mol%) = (G) + (H).

### 2.3. Rheological Analyses

The gel’s rheological behavior was analyzed as previously and conventionally reported for this type of hydrogel [20,26]. Briefly, characterization was carried out using a Physica MCR301 oscillatory rheometer (Anton Paar, Ostfildern Germany) equipped with a parallel-plate geometry, 25 mm plate diameter, 1.0 mm gap, and a Peltier temperature control. Measurements were performed at 37 °C. Amplitude sweep tests were carried out at 37 °C and a frequency of 1.59 Hz over a strain amplitude range of 0.1–100%. G′, G″, and the damping factor values in the linear viscoelastic range (LVR) were derived. The gel rheology was also evaluated after hydration in a physiological medium. Specifically, each gel underwent a series of dilutions in PBS down to HA concentration values in the range of 6–7 mg/mL (attention was paid to avoid excessive dilution, which would result in phase separation). After each dilution, the gels underwent a strain amplitude sweep, as reported above. The G′ and damping factor (G″/G′) values in the LVR were then reported (mean value ± SD) as a function of the HA concentration in the gel.

### 2.4. Sensitivity to Enzymatic Hydrolysis

Sensitivity to enzymatic hydrolysis was studied as previously reported, with slight modifications [20,26]. Briefly, each gel was diluted to 4 mg/mL in PBS and incubated with BTH (5 U/mL) at 37 °C under stirring. After 3 h of incubation, the enzyme was inactivated by boiling the sample for 10 min. The samples were then centrifuged at 13,000 rcf for 5 min. The supernatant was removed and filtered using a pore size of 0.22 μm. The filtered sample (containing only water-soluble HA) was then opportunely diluted in water to measure the HA content (water-soluble HA_BTH_) with a carbazole assay. 

The amount of soluble HA that was already in the commercial gels (water-soluble HA_0_) was measured as previously described [20,26]. Briefly, the same procedure as above, except for the addition of the enzyme, was carried out. 

The amount of HA that was solubilized due to the action of BTH was calculated as:

(1) $$\mathrm{HA}\;\left(\frac{\mathrm{mg}}{\mathrm{mL}}\right)\mathrm{solubilized}\;\mathrm{due}\;\mathrm{to}\;\mathrm{BTH}\;\;=\left({\mathrm{water}\;\mathrm{soluble}\;\mathrm{HA}}_{\mathrm{BTH}}\;\left(\frac{\mathrm{mg}}{\mathrm{mL}}\right)\right)\;-\left({\mathrm{water}\;\mathrm{soluble}\;\mathrm{HA}}_{0}\;\left(\frac{\mathrm{mg}}{\mathrm{mL}}\right)\right)$$

The HA (mg/mL) solubilized due to BTH was considered as a measure of the gels’ sensitivity to enzymatically catalyzed hydrolysis. For each gel, at least four replicates were analyzed. Statistical analyses were performed as indicated in the following. 

### 2.5. Statistical Analyses

Data were statistically evaluated by running one-way ANOVA tests, followed by post hoc tests using Holm correction for multiple comparisons. The level of significance was fixed at 0.05. 

## 3. Results

### 3.1. NMR Analyses

The quantitative data that were derived from ^1^H-NMR and ^13^C-NMR spectra (Figure 2 and Supporting Information) as described in Section 2.2 [5] are reported in Table 1. 

The BDPE/HA_d_ (mol%) values calculated from the ^1^H-NMR analyses (A) varied over a wide range. Namely, the total BDPE/HA_d_(mol%) spanned from a very low amount (2.4%), which was recorded for R_L_, up to around 31%, which was recorded for A_SV_. Similar intermediate values of around 7–8% were found for both T_RHA4_ and J_V_. When considering the BDPE/HA weight ratio (E), the values ranged from 1.2 (R_L_) to 16.0 wt% (A_SV_), with T_RHA4_ and J_V_ still showing similar results (3.4–3.9 wt%). The total BDPE amount (wt%) in the gel (F) was in the range of 0.02–0.4 wt%. 

The ^13^C-NMR spectra allowed us to quantify the BDPE pendant/HA_d_ molar ratio (B) and, therefore, the BDPE bridging/HA_d_ (mol%) (C) and the BDPE bridging/BDPE (mol%) values (D). The data indicate that BDPE that effectively crosslinked two disaccharide units varied from 4.2 to 28.8% compared to the total BDPE amount, with the highest value recorded for T_RHA4_ (D). Therefore, most of the BDDE-derived functionalizations were present on HA chains as pendant groups, rather than as crosslinking moieties. 

The amount (mol%) of crosslinked HA disaccharide units (G) and the amount of branched HA_d_ (mol%) (H) are also reported. Finally, the amount (mol%) of HA_d_ that was chemically modified by BDPE, which describes the total HA modification level (crosslinked + branched) (I), is indicated. A_SV_ turned out to be the most crosslinked gel (6.0 mol% of crosslinked-HA_d_ (G)) and the product that presented the highest percentage of branched disaccharide units (28.5 mol%) (H). It also showed the highest overall modification degree, with 34.5% of its disaccharide units being modified by BDPE (I). 

R_L_ was the least modified gel, with only 2.5% of the HA_d_ being modified. T_RHA4_ and J_V_ showed intermediate HA modification degrees that were closer to that of T_RHA4_, and they turned out to be slightly less modified (modified HA_d_ equal to 8.5 and 9.0 mol%, respectively). However, the crosslinking degree was higher for T_RHA4_, while the amount of branched HA_d_ was greater for J_V_. 

### 3.2. Rheological Analyses and Correlations with HA Modification Parameters

**Figure 3:**
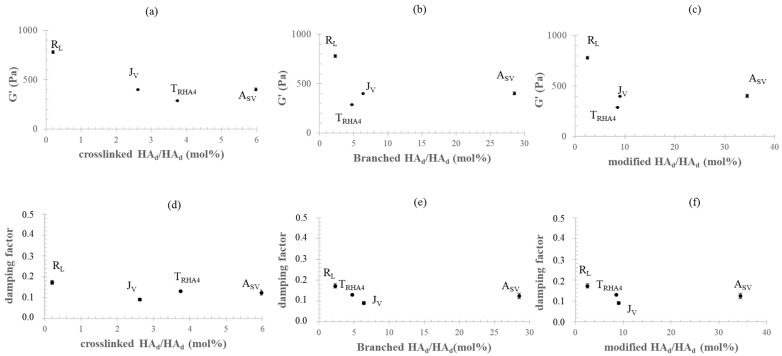
G′ and damping factor for the commercial gels as a function of the HA chemical modification parameters. G′ (**a**–**c**) and damping factor (**d**–**f**) values as a function of the amounts of crosslinked, branched, and modified HA_d_ (mol%).

Figure 3 shows the G′ and damping factor values measured for the gels, which are reported as a function of the amounts (mol%) of crosslinked HA_d_ and branched HA_d_ and as a function of the total amount (mol%) of HA_d_ modified by BDPE. 

Surprisingly, the rheological parameters were not correlated with the biopolymer crosslinking degree the branched HA amount, or the total degree of modification (Figure 3a–f). The data indicated that the least crosslinked gel (R_L_) behaved as the most rigid (Figure 3a). Although turned out to be 2.3-fold more crosslinked than J_v_, A_SV_ showed comparable G′ values. When compared to R_L_, A_SV_ presented a 30-fold higher crosslinking, but was almost twofold less stiff. Even the loss tangent values, which were expected to decrease with increasing crosslinking, did not show any proportional variation with the HA chemical modification parameters (Figure 3d–f).

**Figure 4:**
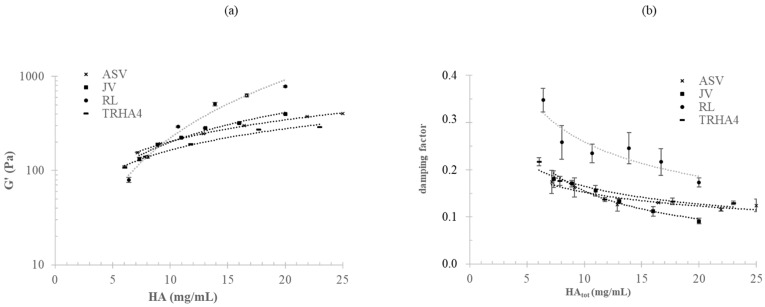
Variation of the rheological parameters of the gels while hydrating. The G′ (**a**) and tan delta (**b**) values measured for the gels with increasing dilution in a physiological medium are plotted as a function of HA concentration.

The G′ and damping factor values were then studied as a function of the progressive dilution of the gels in a physiological medium. The results are shown in Figure 4. 

G′ decreased with the decrease in HA concentration (a), while tanδ increased with progressive gel dilution (b). Both G′ and the damping factor varied as a function of HA concentration, following a power law relation. The dependence of G′ and the loss tangent on HA concentration (C_HA_) was differently marked for the various gels. R_L_ showed the most marked increase in G′ with C_HA_ (G′ = 2.01 (C_HA_)^2.04^; R^2^ = 0.98), followed by J_V_ (G′ = 17.7 (C_HA_)^1.05^; R^2^ = 0.98), and then by A_SV_ and T_RHA4_, which showed the lowest and similar dependences (G′ = 34.9 (C_HA_)^0.77^; G′ = 28.99 (C_HA_)^0.75^). Similarly, the variation of the damping factor with dilution was more marked for R_L_ and J_V_ compared to A_SV_ and T_RHA4_. Overall, the data indicated that the rankings in stiffness and damping factor for the gels were extensively affected by hydration. 

It is worth mentioning that the mechanical spectra of the preparations as they were in the syringe and those of the same gels after the various dilutions tested showed no or a very slight dependence of the moduli on frequency (with G′ being much higher than G″), thus indicating the gel-like behavior of the gels even after dilution (Figure S2). 

**Figure 5:**
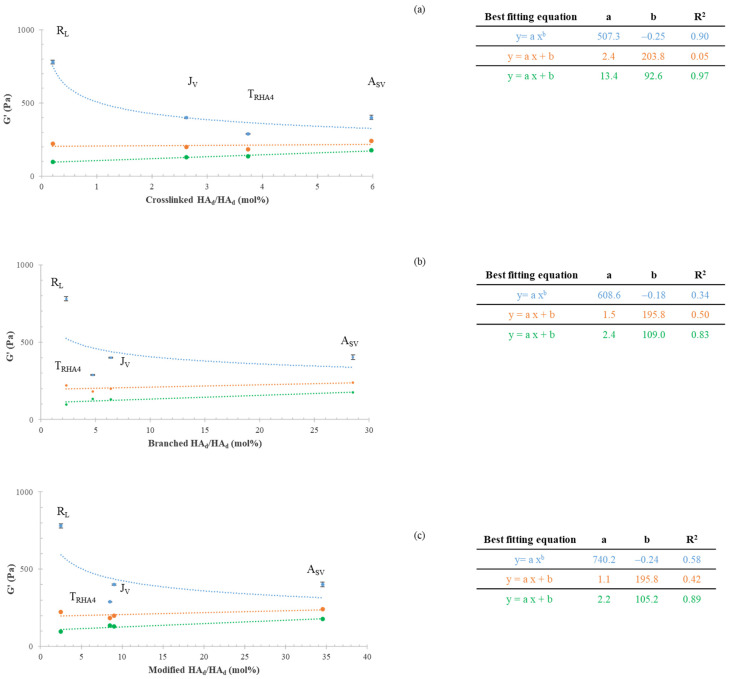
Correlation of G′ with the HA chemical modification parameters of the gels while hydrating. G′ as a function of crosslinked HA_d_ (%) (**a**), branched HA_d_ (%) (**b**), and modified HA_d_ (mol%) (**c**) with increasing hydration in a physiological medium. Specifically, the curves for the G′ values measured for the gels in the syringe (blue) and after twofold (orange) and threefold (green) hydration in the physiological medium are shown. The equation for the best-fitting curve and the related parameters are also shown for each set of experimental data.

Figure 5 shows G′ in relation to the HA modification parameters during gel hydration.

Specifically, the G′ values for the gels as commercialized (blue curves) and for the same gels after twofold (orange curves) and threefold hydration (green curves) in the physiological medium are reported as a function of the amounts of crosslinked HA_d_ (mol%) (a) and branched HA_d_ (mol%) (b) and as a function of the total HA modification (mol%) level (c). The regression with the highest R^2^ value was used to fit each set of data. When considering the gels as commercialized (blue curves), the experimental data were better predicted by a power law model, and the R^2^ values varied from 0.34 to 0.90, with the highest value being found for the G′ vs. crosslinked HAd (mol%) relation. However, the variation of G′ with the crosslinking degree was the opposite of what was expected. Following hydration, the trend completely changed, with G′ increasing with the increase in HA modification, as expected. The goodness of the mathematical prediction of the data also varied with gel dilution. After threefold hydration (green curves), a linear regression better fit the experimental data, with the best correlation being found for G′ vs. crosslinked HA (mol%) (R^2^ equal to 0.97). 

The correlation of G′ with the amount of branched HA_d_ (mol%) and with the total HA modification level (BDPE-modified HA_d_ mol%) also improved with hydration, but lower R^2^ values (0.85–0.89) were reached (Figure 5b,c, green curves). 

The data in Figure 5 also show that the differences in stiffness among the samples were greatly reduced after hydration. For the commercial formulations, G′ was in the range of 280–780 Pa, with the most rigid gel (R_L_) being 2.7-fold stiffer than the most deformable one (T_RHA4_). The threefold-hydrated gels showed G′ values in the range of 97–177 Pa, with the stiffest gel (A_SV_) being only 1.8-fold more rigid than the most deformable sample. 

**Figure 6:**
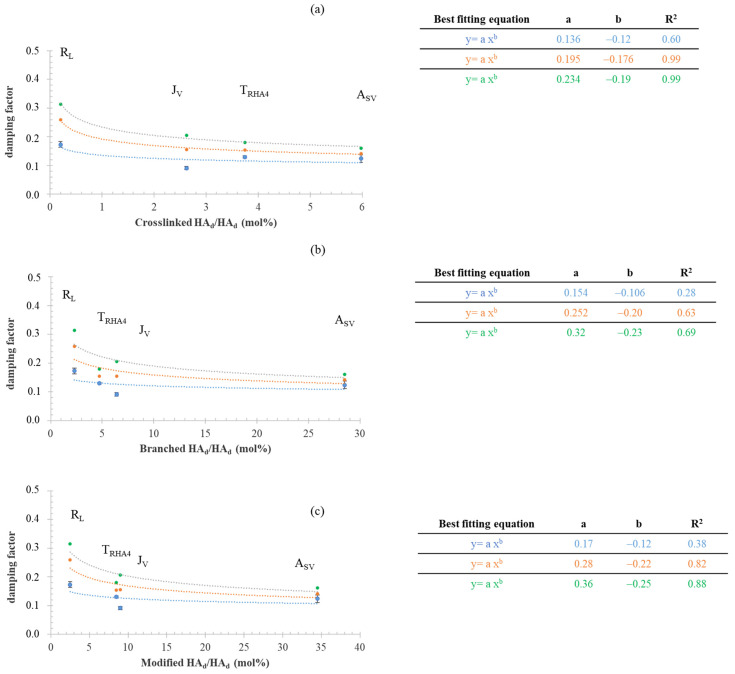
Correlation of the damping factor with the HA chemical modification parameters of the gels while hydrating. Tan delta as a function of crosslinked HA_d_ (%) (**a**), branched HA_d_ (%) (**b**), and modified HA_d_ (mol%) (**c**) with increasing hydration in the physiological medium. Specifically, the curves for the damping factor values measured for the gels in the syringe (blue) and after twofold (orange) and threefold (green) hydration in the physiological medium are shown. The equation for the best-fitting curve and related parameters are also shown for each set of experimental data.

The damping factor values are reported as a function of crosslinked HA_d_, branched HA_d_, and modified HA_d_ (branched + crosslinked HA_d_) in Figure 6a–c, respectively. The blue, orange, and green curves were obtained for the commercial samples and for the samples after two- and threefold hydration, respectively (Figure 6).

A power law regression was used to fit the data showing the highest values for the determination coefficient. The latter improved with hydration, and, for the hydrated gels, the damping factor was strongly correlated with the amount (mol%) of crosslinked HA_d_ (R^2^ 0.99). Even for tan δ, the correlation with the amount of branched HA_d_ and with the total HA modification degree was worse. Unlike G′, the damping factor varied over a wider range after hydration (0.12–0.17 for the gels as commercialized and 0.16–0.31 for the hydrated gels). 

### 3.3. Sensitivity to Enzymatic Hydrolysis and Correlation with HA Modification Parameters

The gels’ sensitivity to enzymatic action was evaluated by monitoring the amount (mg/mL) of biopolymer that was solubilized due to incubation with BTH (5 U/mL; 3 h; 37 °C). The results are reported in Table 2. All of the samples tested were shown to have sensitivity to BTH. Different levels of solubilization extents varying from 1.1 ± 0.2 to 2.8 ± 0.5 mg/mL were recorded.

The gels presenting an amount of 2.6–6.0% crosslinked HA_d_ (mol%) showed comparable degrees of solubilization (1.1–1.6 mg/mL, *p* > 0.05), while a significantly higher degree of solubilization (2.8 ± 0.5 mg/mL; *p* < 0.05) was recorded for the least crosslinked gel (0.2 mol% crosslinked HA_d_/HA_d_) under the same conditions. When considering solubilization due to BTH in relation to the total HA modification degree, the data indicated that variation in the amount of BDPE-modified HA (mol %) in the range of 8.5–34.5 (mol%) did not result in different levels of stability with respect to enzymatic action, while lowering the level of derivatization down to 2.5% led to worse stability. 

## 4. Discussion

In the wide framework of studies on HA–BDDE hydrogels for dermo-aesthetic applications, we aimed to shed light on the correlation between hydrogels’ rheology and stability and their HA crosslinking/modification parameters. 

Four clinically available HA–BDDE dermal fillers were investigated for this purpose. The BDPE/HA_d_ molar ratio values (Table 1 (A)) derived from ^1^H-NMR analyses fell within the range reported in the literature for similar gels, except for those of A_SV_, which presented a higher BDPE/HA_d_ content [3,9,12,20]. It is noteworthy that the BDPE/HA_d_ molar ratio varied over a wide range (2–31%) even though the same clinical indications were given for the hydrogels tested.

Interesting chemical aspects that were scarcely considered before for these hydrogels emerged from the ^13^C-NMR analyses. At first, the data indicated that most (more than 70%) of the BDPE bonded to HA did not lead to HA crosslinking, but to HA bearing BDPE appendages. Discrimination between the two BDPE functionalities (bridging or pendant) allowed for the calculation of the real total HA modification level (I) [5]. The latter did not simply correspond to the BDPE/HA_d_ molar ratio (A), as is commonly determined for these products via ^1^H-NMR. In fact, each BDPE-bridging moiety was responsible for two chemically modified HA disaccharide units; therefore, a higher total HA modification (I) level was expected in comparison to the BDPE/HA_d_ ratio (A). However, due to the low number of BDPE-bridging functionalities, only a slight difference was recorded between the (A) and (I) parameters (Table 1). It is noteworthy that the (I) values were in the range of 2.5–34.5%, while a total HA modification of 4–63% would be expected in the case of 100% BDPE bridging. 

As very few studies in the literature have reported, our data indicated that attention must be paid when ranking the gels in terms of their HA modification parameters that are based only on the BDPE/HA_d_ ratio [3,12,19,31]. In fact, since the bridging BDPE/total BDPE ratio greatly varied from one product to another (Table 1 (D)), the rankings in the HA crosslinking degree (G) can be variously marked or even completely different compared to those for the BDPE/HA ratio (A) or total HA modification level (I). For instance, for the products analyzed here, the most derivatized gel (A_SV_) showed a 4.4-fold higher BDPE/HA_d_ ratio than that of J_V_ (Table 1 (I)), but it was only 2.3-fold more crosslinked (Table 1 (G)). Further, J_V_ showed a higher BDPE/HA_d_ amount than that of T_RHA4_, but was less crosslinked. 

The samples tested presented very similar concentrations of water-insoluble HA–BDDE fractions, which are mainly responsible for the typical elastic behavior of these gels. Therefore, an increase in the rheological properties with the level of HA crosslinking and/or total HA modification was expected. Hence, the absence of a correlation between the G′ and damping factor values and the biopolymer modification parameters (crosslinked HA_d_; branched HA_d_; modified HA_d_) was surprising. The trend found for G′ and the loss tangent vs. the amount of crosslinked-HA_d_ was very far from what was expected (Figure 3). These findings mainly suggested that, aside from the network’s crosslinking density, other parameters play a role in gels’ relative rheology. A certain effect related to the diversity in the water-soluble fractions and to the potential differences (limited differences considering the specific reaction conditions) in the lengths of the chains undergoing crosslinking can be expected. The extent of gel hydration was rationally considered and investigated as the other potentially highly impacting factor. 

It is well known that the HA networks in the commercial formulations do not exploit their maximum hydration capabilities and that gels are further hydrated after being delivered into the dermis or subdermis. Notwithstanding this, the data on HA–BDDE gels reported so far refer only to the rheological properties of the gels as they are in the syringe, with the study by Ilyin et al. being the only one to consider the gels’ rheological parameters after fivefold dilution [6]. Here, this latter aspect was further explored. The gels were progressively diluted in a physiological medium to simulate hydration in vivo while monitoring changes in their rheological behavior. The variation of G′ and the damping factor with decreasing HA concentration (Figure 4) was predictable. However, it was remarkable that the dilution affected the gels to different extents due to the specific features of the network. Specifically, the data indicated a more marked variation in the rheology with dilution for less crosslinked hydrogels. As a consequence, the rankings in mechanical behavior completely changed after hydration. Finally, for the hydrated gels, the G′ and damping factor values were correlated with the crosslinking level, as expected (Figure 5). The less significant correlation found with respect to the other HA modification parameters suggested that the tuning of HA crosslinking—more than HA branching—is key for adjusting the mechanical behavior of a gel once it is equilibrated in the tissue. Overall, the data collected here indicate that, for the gels in the syringe, the level of (partial) hydration used (HA concentration) plays a key role in their rheology, while, after hydration, their mechanical behavior is mainly driven by the extent of HA crosslinking. This is clearly represented, for instance, by the R_L_ data: notwithstanding that it had the lowest crosslinking/modification degree, R_L_ behaved as the most rigid product in its commercial form (Figure 5). However, when allowed to hydrate, it markedly lost its rigidity, finally behaving as the most deformable sample (lowest G′), which was consistent with its low crosslinking degree (Figure 5). The combination of the specific HA crosslinking level and the HA content in 1 mL of the commercial gel resulted, for A_SV_, in an interesting performance. Based on the collected data, a higher projection/volumizing ability (highest G′ and lowest damping factor) was expected for A_SV_ after being equilibrated in the tissue, while the lower G′ recorded for this gel in the syringe suggested an ease of delivery that would be similar to or higher than that of the other samples. It is also worth noting that a quantification of the effect of the soluble fraction (concentration and molecular weight) on the rheology of the formula would be valuable for furthering tailoring the injectability of the final preparation. 

Further, the data highlighted a key point that impacted the characterization of these hydrogels in relation to the prediction of in vivo performance. To date, the relative gel projection capacity has been predicted by comparing the rheological parameters of commercial samples [10,14,15,16,18,20,24,26]. Based on our findings, these parameters are representative of gel’s behavior only during delivery and immediately after the delivery to the dermis, but they do not characterize the gels once they have equilibrated in the tissue. Therefore, hereafter, more attention should be paid to the characterization of gels’ rheological features, which may be more predictive when they are also evaluated for hydrated gels. Comparing the rheology of the formulas to be injected only provides evidence on their relative injectability. 

Useful information on the relation between gel stability with respect to hyaluronidases and HA structural modification was also provided. The derivatization of HA by BDPE (not only crosslinking, but also functionalization with pendant moieties) is expected to affect the recognition of the biopolymer by the enzyme, thus increasing gel stability and, therefore, prolonging action in vivo. The collected data confirmed what was expected only for degrees of derivatization/crosslinking up to 8-9/3-4 mol%, and they showed that there was no significant effect on BTH activity for higher levels of HA modification. This would suggest that the conformational variation in HA occurring in the lower range of modification is more significant for the recognition of the biopolymer by the enzyme. A further advance in the understanding of the dependence of HA–BDDE gels’ stability on HA modification may be achieved when investigating whether the increase in crosslinking—while keeping the BDPE/HA ratio constant—influences sensitivity to hyaluronidases or not. 

## 5. Conclusions

For the first time, HA–BDDE gels that were intended as dermal fillers were analyzed to investigate how the formulations’ rheology and stability related to their HA chemical modification parameters. Rheological data were also provided for the gels while being hydrated in a physiological medium (to mimic the gels’ equilibration in tissue). The gels greatly differed in terms of the levels of total HA derivatization and the functionalities of branched HA and HA crosslinked by BDPE, with branching representing the main type of modification. 

The rheological properties of the hydrated gels were correlated well with the level of HA crosslinking, while the mechanical behavior of the samples as commercialized was mainly controlled by the HA concentration. An unexpected and strong effect of hydration on the gels’ ranking in terms of rheology was demonstrated, with implications for the characterization of these products. The results indicated that there was no improvement in the gels’ stability with respect to enzymatic hydrolysis for derivatization and crosslinking levels higher than 9 and 3 mol%, respectively.

Overall, the collected data are of interest for the design, in vitro characterization, and prediction of the performance of these types of hydrogels.

## Figures and Tables

**Table 1:** Results from the NMR analyses.

Sample	(A)	(B)	(C)	(D)	(E)	(F)	(G)	(H)	(I)
	BDPE Total/HA_d_ (mol%)	BDPE Pendant/HA_d_ (mol%)	BDPE Bridging/HA_d_ (mol%)	BDPE Bridging/ BDPE-Total (mol%)	BDPE/HA (wt%)	BDPE in the Gel (wt%)	Crosslinked HA_d_/HA_d_ (mol%)	Branched HA_d_/HA_d_ (mol%)	Modified HA_d_/HA_d_ (mol%)
A_SV_	31.5	28.5	3.0	9.5	16.0	0.40	6.0	28.5	34.5
T_RHA4_	6.6	4.7	1.9	28.8	3.4	0.08	3.7	4.7	8.5
J_V_	7.7	6.4	1.3	16.9	3.9	0.08	2.6	6.4	9.0
R_L_	2.4	2.3	0.1	4.2	1.2	0.02	0.2	2.3	2.5

BDPE/HA_d_ (mol%): BDPE (mol)/HA_d_ (mol) × 100. BDPE/HA_d_ (wt%): BDPE (mg)/HA (mg) × 100. BDPE in the gel (wt%): BDPE (mg)/gel (mg) × 100. BDPE pendant/HA_d_ (mol%): BDPE pendant (mol)/HA_d_ (mol) × 100. BDPE bridging/HA_d_ (mol%): BDPE bridging (mol)/HA_d_ (mol) × 100. BDPE bridging/BDPE (mol%): BDPE bridging (mol)/BDPE (mol) × 100. Crosslinked HA_d_/HA_d_ (mol%): HA_d_ modified by BDPE bridging (=BDPE bridging (mol)×2)/HA_d_ (mol)×100. Branched HA_d_/HA_d_ (mol%): HA_d_ modified by BDPE pendant (=BDPE pendant (mol))/HA_d_ (mol) × 100. Modified HA (mol%): [crosslinked-HA_d_ (mol) + branched-HA_d_ (mol)]/HA_d_ (mol) × 100, where HA_d_ stands for “HA disaccharide unit”. The parameters in (A)–(I) were derived as reported in Section 2.2.

**Table 2:** Amount (mg/mL) of HA hydrogel solubilized due to 3 h of incubation with bovine testicular hyaluronidase (5 U/mL). The HA modification parameters derived from the NMR analyses are also reported for the sake of readability. * *p* > 0.05; *p* < 0.05 compared to A_SV_, T_RH4_, and J_V_.

Filler	HA(mg/mL) Solubilized Due to BTH (5 U/mL, 3 h)	Modified HA_d_/HA_d_ (mol%)	Crosslinked HA_d_/HA_d_ (mol%)	Branched HA_d_/HA_d_ (mol%)
A_SV_	1.1 ± 0.2 *	34. 5	6.0	28.5
T_RH4_	1.6 ± 0.4 *	8.5	3.7	4.7
J_V_	1.4 ± 0.2 *	9.0	2.6	6.4
R_L_	2.8 ± 0.5	2.5	0.2	2.3

## Data Availability

All data that are critical for the reader’s understanding and discussion of outcomes are reported within the manuscript or as a supplementary file. Additional raw data are available from the corresponding authors upon request.

## Supplementary Materials

The following supporting information can be downloaded at https://www.mdpi.com/article/10.3390/polym14122402/s1, Figure S1: (a, a′, a″) ^1^H-NMR (400 MHz, 300K, D_2_O) and (b, b′, b″) full and (c, c′, c″) zoomed ^13^C-NMR (400 MHz, 300K, D_2_O) spectra of J_V_, T_HRA4_, and R_L_ samples, respectively. The signals used for the quantitative determinations are indicated.
