# Supplementary figures and images for: Hyaluronan Hydrogels: Rheology and Stability in Relation to the Type/Level of Biopolymer Chemical Modification
Source: Polymers (Basel). 2022 Jun 14;14(12):2402. doi: 10.3390/polym14122402 (PMC9228881; doi:10.3390/polym14122402)

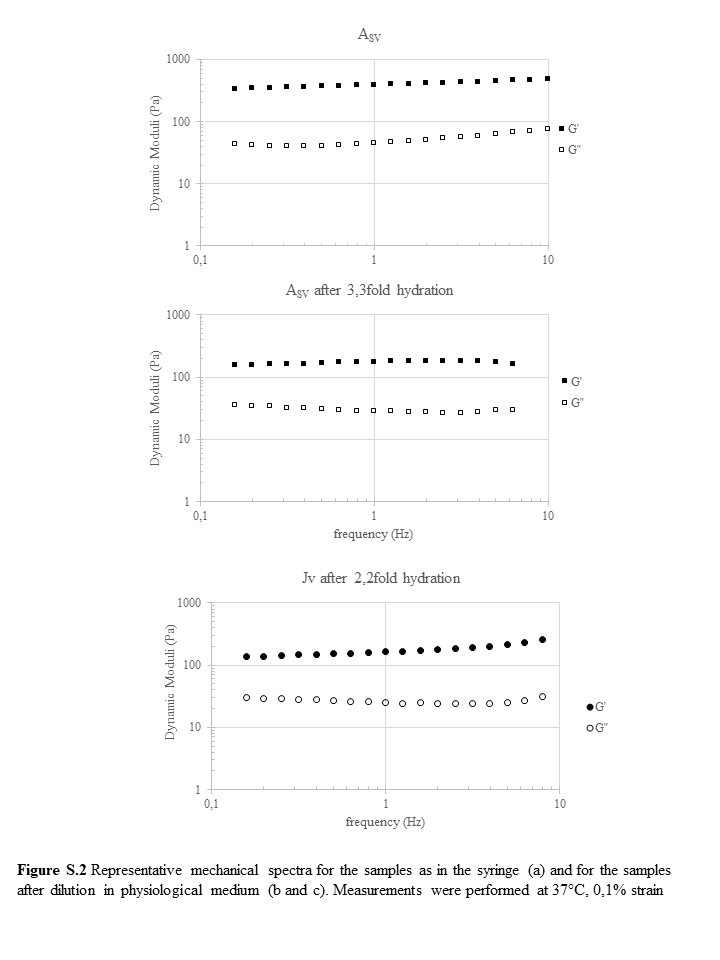

Supplement: Supplementary file 1 [file polymers-14-02402-s001.zip › final files/Figure S.2_300dpi.tif]

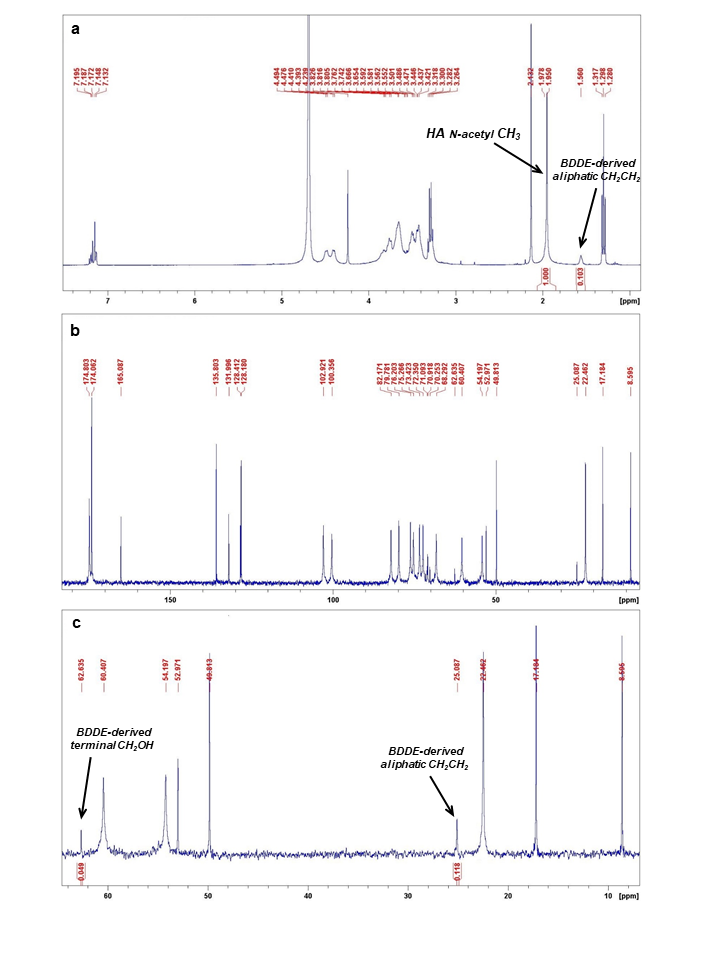

Supplement: Supplementary file 1 [file polymers-14-02402-s001.zip › final files/Figure_S1a_final_1000dpi.tif]

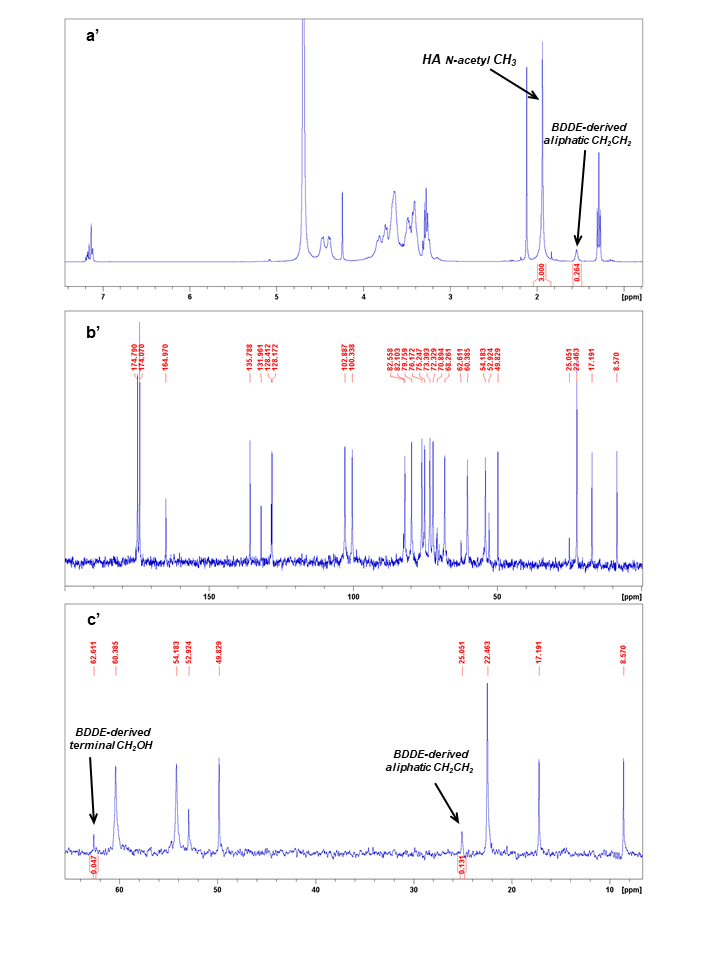

Supplement: Supplementary file 1 [file polymers-14-02402-s001.zip › final files/Figure_S1b_final_1000dpi.tif]

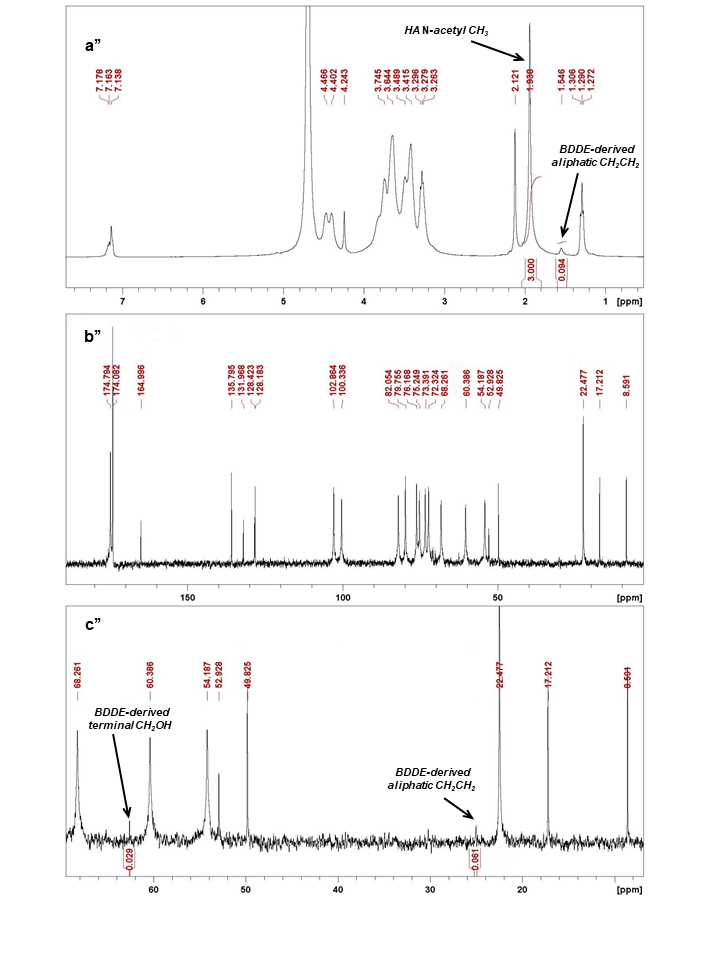

Supplement: Supplementary file 1 [file polymers-14-02402-s001.zip › final files/Figure_S1c_final_1000dpi.tif]
